# NOD2-mediated dual negative regulation of inflammatory responses triggered by TLRs in the gastrointestinal tract

**DOI:** 10.3389/fimmu.2024.1433620

**Published:** 2024-09-30

**Authors:** Sho Masaki, Yasuhiro Masuta, Hajime Honjo, Masatoshi Kudo, Tomohiro Watanabe

**Affiliations:** Department of Gastroenterology and Hepatology, Kindai University Faculty of Medicine, Osaka-sayama, Osaka, Japan

**Keywords:** nucleotide-binding oligomerization domain 2, toll-like receptor, inflammatory bowel disease, nuclear factor- κB, interferon regulatory factor

## Abstract

Loss-of-function mutations in nucleotide-binding oligomerization domain 2 (*NOD2*) constitute the primary risk factors for Crohn’s disease. NOD2 is an intracellular sensor for muramyl dipeptide (MDP), a small molecule derived from the peptidoglycan layer of bacterial cell wall. Although NOD2 is involved in host immune responses, much attention has been paid to the involvement of NOD2 in the maintenance of intestinal homeostasis. Despite the fact that the proinflammatory cytokine and chemokine responses induced by NOD2 activation alone are weaker than those induced by toll-like receptors (TLRs), NOD2 plays a crucial role in host defense against invading pathogens and in the regulation of immune responses. Recent studies have highlighted the importance of negative regulatory functions of NOD2 in TLRs-mediated proinflammatory cytokine responses. MDP-mediated activation of NOD2 induces interferon regulatory factor 4 (IRF4) expression, thereby suppressing nuclear factor-κB-dependent colitogenic cytokine responses through the inhibition of Lys(K)63-linked polyubiquitination on receptor-interacting serine/threonine protein kinase 2. MDP-mediated activation of NOD2 also downregulates TLR9-induced type I IFN responses by inhibiting the K63-linked polyubiquitination of TNF receptor-associated factor 3 via deubiquitinating enzyme A (DUBA) expression. Thus, NOD2 exerts dual negative regulation of TLRs-mediated proinflammatory cytokine and type I IFN responses by inducing the expression of IRF4 and DUBA, respectively. In this review, we summarize the molecular mechanisms whereby NOD2 activation suppresses TLRs-mediated proinflammatory and type I IFN responses. In addition, we discuss the clinical relevance of the NOD2-mediated negative regulation of TLRs in inflammatory bowel disease.

## 1 Introduction

Trillions of bacteria colonize the human gastrointestinal (GI) tract (1). Gut macrophages and dendritic cells (DCs) express functional toll-like receptors (TLRs) and nucleotide-binding oligomerization domain (NOD)-like receptors (NLRs) to detect bacterial cell wall components and nucleic acids to defend the body against invading bacteria (2, 3). However, in the steady state, intestinal commensal bacteria coevolve with the host immune system to create a symbiotic relationship that prevents harmful proinflammatory cytokine responses by macrophages and DCs (1). Thus, gut macrophages and DCs display tolerogenic responses against intestinal commensal bacteria and preserve their ability to mount robust proinflammatory cytokine responses upon encountering pathogens. The downregulation of proinflammatory cytokine responses against commensal intestinal bacteria contributes to the maintenance of intestinal homeostasis.

Inflammatory bowel disease (IBD), chronic relapsing inflammatory disorders of the GI tract, are categorized into Crohn’s disease (CD) and ulcerative colitis (UC) (4, 5). Excessive production of proinflammatory cytokines, such as IL-6, IL-12, IL-23, and TNF-α, due to defective immune tolerance to intestinal commensal bacteria leads to the development of CD and UC (4, 5). Several lines of evidence support the concept that proinflammatory cytokine responses against intestinal bacteria play a crucial role in the immunopathogenesis of IBD. First, the production of proinflammatory cytokines, such as TNF-α, IL-6, IL-12, and IL-23, is enhanced in gut lamina propria macrophages and DCs upon stimulation with TLR ligands in patients with IBD (4, 5). Second, biologics targeting TNF-α, IL-12, and IL-23 have shown remarkable success in the induction and maintenance of remission in both CD and UC (4, 5). Finally, application of genome-wide association studies to CD has led to the identification two major susceptibility genes associated with CD—nucleotide-binding oligomerization domain 2 (*NOD2*) and autophagy-related 16 like 1 (*ATG16L1*) (6–8). Notably, both NOD2 and ATG16L1 are involved in the intracellular processing of bacterial components, and DCs bearing CD-associated *NOD2* or *ATG16L1* mutations produce large amounts of proinflammatory cytokines upon exposure to TLR ligands (6–8). In this review, we summarize the recent insights into the mechanism whereby CD-associated *NOD2* mutations lead to chronic intestinal inflammation through excessive production of proinflammatory cytokines upon exposure to TLR ligands.

## 2 Structure and expression of NOD2

**Figure 1:**
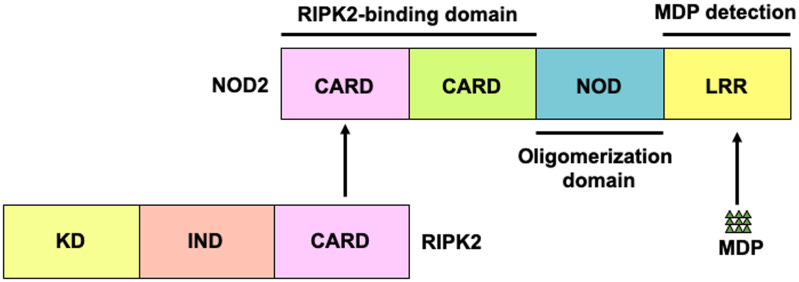
Structure of nucleotide-binding oligomerization domain 2. Nucleotide-binding oligomerization domain 2 (NOD2) is a cytosolic protein that detects muramyl dipeptides (MDP) derived from gram-positive and -negative bacteria. NOD2 comprises two N-terminal caspase recruitment domains (CARDs)—a nucleotide-binding oligomerization domain (NOD) and a leucine-rich repeat (LRR) domain. CARD is a binding domain for receptor-interacting serine/threonine protein kinase 2 (RIPK2). LRR detects MDP.

NOD2 comprises two N-terminal tandem caspase recruitment domains (CARD)—a central NOD domain and a C-terminal leucine-rich repeat (LRR) domain (**Figure 1**) (3, 8, 9). The LRR domain is a ligand-binding domain, and NOD2 recognizes muramyl dipeptide (MDP), a small molecule derived from peptidoglycan (PGN) present in the cell wall of gram-positive and -negative bacteria. The CARD domains are necessary for the interaction between NOD2 and the downstream signaling molecules (3, 8, 9). Receptor-interacting serine/threonine protein kinase 2 (RIPK2) is a critical downstream signaling molecule for NOD2, which interacts with RIPK2 through CARD-CARD interactions (3, 8, 9). Mutations in *NOD2* constitute the major risk factors for CD development, and three major mutations in *NOD2* (Arg702Trp, Gly908Arg, and Leu1007fsinsC) increase the risk by multiple-fold (6, 10). Importantly, macrophages or DCs bearing these CD-associated *NOD2* mutations display defective production of cytokines and chemokines upon stimulation with the NOD2 ligand, MDP (3, 8, 9). Therefore, CD-associated *NOD2* mutations are considered as loss-of-function mutations. Given that NOD2 detects MDP derived from intestinal bacteria, the association between *NOD2* mutations and CD suggests that MDP-mediated activation of NOD2 contributes to the maintenance of intestinal homeostasis through the downregulation of proinflammatory cytokine responses against the gut bacteria. Conversely, excessive proinflammatory cytokine responses against gut bacteria caused by *NOD2* mutations predispose individuals to CD.

NOD2 is a cytosolic protein that is stably expressed primarily in macrophages and DCs (3, 8). In the epithelial cell populations, NOD2 protein expression is limited to Paneth cells (3, 8). However, intestinal epithelial cells express *NOD2* mRNA at a steady state, and its expression is enhanced by proinflammatory cytokines such as TNF-α and IFN-γ (11).

## 3 Signaling pathways of NOD2

**Figure 2:**
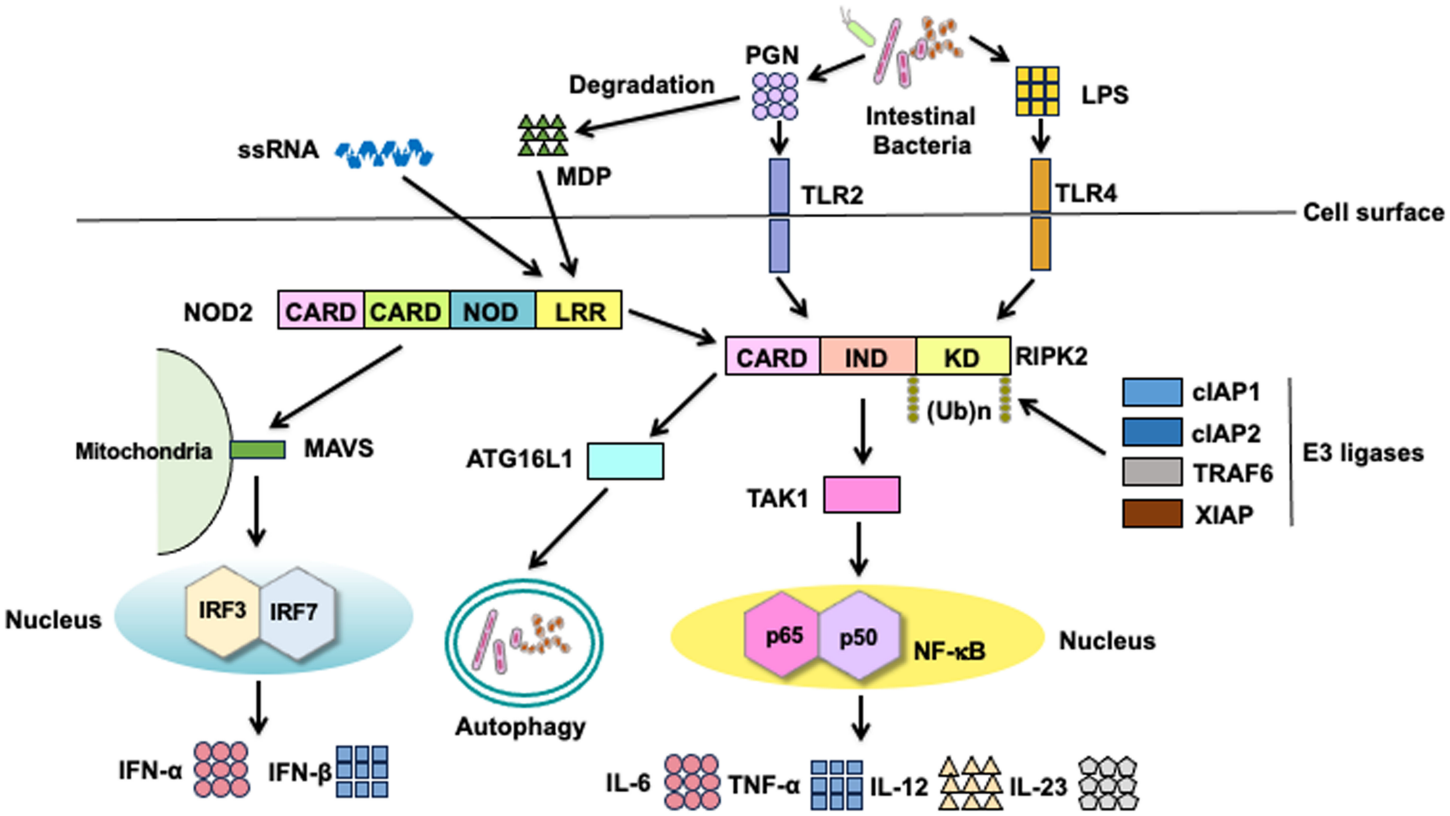
Signaling pathways of nucleotide-binding oligomerization domain 2 and toll-like receptors. Nucleotide-binding oligomerization domain 2 (NOD2) is a cytosolic protein that detects muramyl dipeptides (MDP) derived from gram-positive and -negative bacteria. MDP is a degradation product of the bacterial cell wall component peptidoglycan (PGN). Recognition of MDP by the leucine-rich repeat (LRR) domain leads to the activation of receptor-interacting serine/threonine protein kinase 2 (RIPK2) via a caspase recruitment domain (CARD)-CARD interaction. RIPK2 is subjected to Lys63 (K63)-linked polyubiquitination by E3 ligases, including cellular inhibitor of apoptosis protein 1 (cIAP1), cIAP2, TNF receptor-associated factor 6 (TRAF6), and X-linked inhibitor of apoptosis protein (XIAP). K63-linked polyubiquitination on RIPK2 results in the physical interaction between RIPK2 and TGF-β-activated kinase 1 (TAK1). Activation of TAK1 leads to the nuclear translocation of nuclear factor-κB (NF-κB) subunits to induce the transcription of proinflammatory cytokine genes. In addition to the nuclear translocation of NF-κB subunits, TAK1 mediates the activation of mitogen-activated protein kinases (MAPKs). NOD2 activation results in the recruitment of autophagy-related 16 like 1 (ATG16L1) to induce autophagy. Cell surface toll-like receptor 2 (TLR2) and TLR4 recognize PGN and lipopolysaccharide (LPS), respectively. RIPK2 is a downstream signaling molecule shared by NOD2, TLR2, and TLR4. Activation of RIPK2 by TLR2 and TLR4 causes nuclear translocation of NF-κB subunits to induce the transcription of proinflammatory cytokine genes. In addition to NF-κB activation, NOD2 mediates the production of type I IFNs upon sensing of single-stranded RNA (ssRNA) and interaction with mitochondrial antiviral signaling protein (MAVS).

Classical signaling pathways of NOD2 depend on RIPK2 activation (**Figure 2**) (3, 8). Detection of MDP by NOD2 results in the activation of nuclear factor-κB (NF-κB) and mitogen-activated protein kinases (MAPKs), which ultimately leads to the transcription of proinflammatory cytokine and chemokine genes. Notably, the synthesis of proinflammatory mediators induced by NOD2 activation is much lower than that induced by TLRs activation (3, 8). Nonetheless, NOD2 plays a crucial role in host defense against invading pathogens and in the regulation of immune responses. Recognition of MDP by intracellular NOD2 immediately activates RIPK2 through a CARD-CARD interaction (3, 8, 12). The macrophages isolated from RIPK2-deficient mice exhibit defective NF-κB-dependent cytokine responses upon stimulation with MDP; this indicates that RIPK2 functions as a critical signaling molecule in the NOD2-mediated signaling pathways (13, 14). In the aforementioned previous studies, the production of NF-κB-dependent cytokines mediated by TLRs was comparable between the RIPK2-deficient and RIPK2-intact mice, suggesting that RIPK2 activation is not involved in the TLRs-mediated cytokine responses (13, 14). However, whether TLRs utilize RIPK2 as a downstream signaling molecule remains controversial. RIPK2 activation is involved in the TLRs-mediated proinflammatory cytokine responses (15–18). Indeed, interactions between RIPK2 and TLR2 or TLR4 have been reported; macrophages derived from RIPK2-deficient mice produce reduced amounts of IL-6 and TNF-α upon stimulation with TLR2 and TLR4 ligands when compared with those from RIPK2-intact mice (15–18). Thus, RIPK2 functions as a downstream signaling molecule not only for NOD2 but also for TLRs. The balance between the NOD2-RIPK2 and TLRs-RIPK2 axes is critical for the maintenance of intestinal homeostasis (See subsequent sections).

RIPK2 activation is tightly regulated by polyubiquitination regardless of whether RIPK2 interacts with NOD2 or TLRs (12, 19–23). Polyubiquitination is a posttranslational modification involved in immune responses, especially the NF-κB signaling pathway (24, 25). Lys63 (K63)- and K48-linked polyubiquitination of RIPK2 induces the activation and degradation of RIPK2, respectively, in signaling pathways. Thus, RIPK2-mediated NF-κB activation depends upon K63-linked polyubiquitination conjugated by various E3 ligases, including cellular inhibitor of apoptosis protein 1 (cIAP1), cIAP2, TNF receptor-associated factor 6 (TRAF6), X-linked inhibitor of apoptosis protein (XIAP), and pellino3 (**Figure 2**) (12, 16, 19–23, 26). In addition to the RIPK2 activation by K63-linked polyubiquitination, recent studies have revealed the involvement of methionine1 (Met1)-linked linear polyubiquitination by the linear ubiquitin chain assembly complex (LUBAC) in the interaction between RIPK2 and XIAP (19, 22). These previous studies established that K63-linked and Met1-linked polyubiquitination on RIPK2 is an indispensable step for the RIPK2-mediated activation of NF-κB followed by proinflammatory cytokine responses (**Figure 2**). TGF-β-activated kinase 1 (TAK1) recruits RIPK2 and subsequently induces the nuclear translocation of NF-κB subunits (3, 8). NF-κB activation by NOD2 is critical for the maintenance of intestinal homeostasis. MDP-mediated activation of NOD2 induces the activation of interferon regulatory factor 4 (IRF4) and ATG16L1, both of which inhibit the polyubiquitination of RIPK2, thereby reducing the NF-κB-dependent proinflammatory cytokine responses against TLR ligands derived from intestinal bacteria (27–29). In other words, the defective activation of IRF4 and ATG16L1 due to CD-associated *NOD2* mutations leads to excessive NF-κB-dependent cytokine responses upon exposure to TLR ligands.

IRF3 and IRF7 are vital transcription factors for the production of type I IFNs (30). Activation of endosomal TLRs (TLR3, TLR7, and TLR9) and TLR4 leads to the robust production of type I IFNs via the nuclear translocation of IRF3 and IRF7 (2). In addition to MDP, NOD2 recognizes viral single-stranded RNA (ssRNA) to induce type I IFN production (**Figure 2**) (31). Binding of ssRNA to NOD2 enables its interaction with mitochondrial antiviral signaling protein (MAVS), followed by the nuclear translocation of IRF3 (31). Importantly, the ssRNA-mediated activation of NOD2 induces type I IFN responses in an RIPK2-independent manner.

Intracellular proteins derived from organelles and microorganisms are subjected to degradation (32). This process is known as autophagy, an essential homeostatic process whereby cells digest their own components to adapt to nutrient deprivation (32). Autophagy also contributes to the digestion of microorganisms and processing of antigens for antigen presentation by macrophages and DCs (32). Loss-of-function mutations in *ATG16L1* are associated with CD development (6–8). Although ATG16L1 was initially discovered as a molecule involved in autophagy, it has been shown to function as a signaling molecule for NOD2 and TLRs (7). Importantly, the MDP-mediated activation of NOD2 induces a physical interaction between NOD2 and ATG16L1 to promote autophagy and antigen presentation (**Figure 2**) (33, 34). Cooney et al. reported that DCs bearing CD-associated *NOD2* or *ATG16L1* mutations exhibit defective autophagic responses upon exposure to intestinal bacteria (34). Although NOD2-mediated autophagic responses depend on the recruitment of ATG16L1, it remains controversial whether such autophagic responses involve RIPK2 activation (33, 34). However, ATG16L1 downregulates the RIPK2-mediated NF-κB activation by inhibiting the K63-linked polyubiquitination of RIPK2 (35, 36), suggesting the existence of an interaction between ATG16L1 and RIPK2.

Collectively, the NOD2 signaling pathways participate in diverse immunological responses through the activation of NF-κB, IRF3/7, and autophagy. Recognition of MDP derived from intestinal bacteria contributes to the maintenance of intestinal homeostasis through the induction of negative regulators of TLRs and the promotion of autophagic responses, whereas failure to operate such protective mechanisms due to defective NOD2 signaling leads to the development of CD. This has been described in the following section.

## 3 NOD2 mutations and CD

To date, three mechanisms have been proposed to explain the development of CD as a result of loss-of-function mutations in *NOD2* (8, 37). The first mechanism focuses on the function of Paneth cells, located in the crypts of Lieberkühn of the small intestine. Upon recognizing MDP derived from intestinal bacteria, Paneth cells expressing NOD2 constitutively produce α-defensin, a prototypical antimicrobial peptide (8, 37). Accordingly, loss of NOD2 function results in decreased production of α-defensin by Paneth cells, which in turn leads to defective host defense against bacteria. Indeed, NOD2-deficient mice exhibit defective host defense against certain bacteria, and patients with CD bearing *NOD2* mutations display decreased production of α-defensin when compared to those with intact *NOD2* gene (38, 39). However, another study has shown that reduced expression of α-defensin is independent of the *NOD2* mutation status (40). The second mechanism focuses on autophagy induced by the MDP-mediated activation of NOD2. The recognition of MDP derived from intestinal bacteria by NOD2 results in the induction of autophagy-mediated bactericidal effects in an ATG16L1-dependent manner, as mentioned above (33, 34). Thus, the loss of NOD2 function leads to defective autophagic responses owing to the defective interaction between NOD2 and ATG16L1. These defective autophagic responses in the presence of loss of function *NOD2* mutations lead to excessive proinflammatory responses in response to the increased burden of gut bacteria (33, 34). The third mechanism focuses on the NOD2-mediated negative regulation of inflammatory responses triggered by TLRs (8, 37). The MDP-mediated activation of NOD2 negatively regulates TLRs-mediated proinflammatory cytokine responses, and the presence of CD-associated *NOD2* mutations increases the risk of intestinal inflammation due to excessive production of proinflammatory cytokines mediated by TLRs. The third mechanism has been discussed in the subsequent sections.

### 3.1 NOD2-mediated negative regulation on the production of NF-κB-dependent proinflammatory cytokines triggered by TLRs

**Figure 3:**
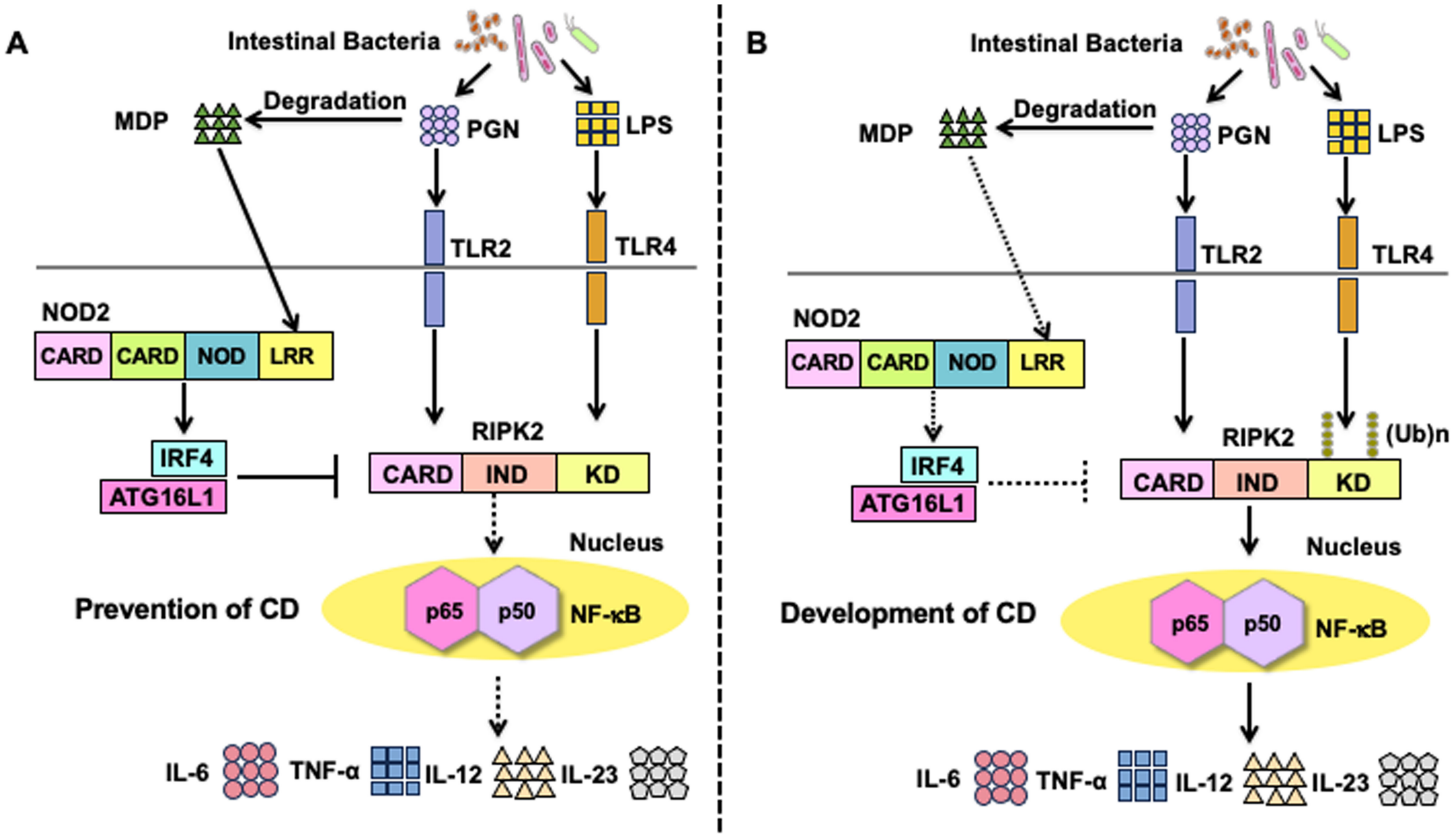
Molecular mechanisms underlying the negative regulation on toll-like receptors-mediated proinflammatory cytokine responses by the activation of nucleotide-binding oligomerization domain 2. **(A)** Molecular mechanisms underlying the negative regulation of toll-like receptors (TLRs)-mediated inflammatory responses in the presence of intact nucleotide-binding oligomerization domain 2 (NOD2). Muramyl dipeptide (MDP) activation of NOD2 enhances the expression of interferon regulatory factor 4 (IRF4). IRF4 acts synergistically with autophagy-related 16 like 1 (ATG16L1) to inhibit the Lys63 (K63)-linked polyubiquitination of receptor-interacting serine/threonine protein kinase 2 (RIPK2). As a result, TLR2 or TLR4-mediated activation of nuclear factor-κB (NF-κB) followed by the production of proinflammatory cytokines (TNF-α, IL-6, IL-12, and IL-23) is markedly suppressed. Downregulation of NF-κB-dependent cytokine responses (TNF-α, IL-6, IL-12, IL-23) contributes to the maintenance of tolerogenic responses toward intestinal bacteria, thereby inhibiting the development of Crohn’s disease (CD). **(B)** Molecular mechanisms underlying CD development in the presence of *NOD2* mutations. CD-associated *NOD2* mutations are loss-of-function mutations that fail to recognize MDP. In the presence of CD-associated *NOD2* mutations, expression of IRF4 or activation of ATG16L1 is not induced. NF-κB-dependent proinflammatory cytokine responses (TNF-α, IL-6, IL-12, and IL-23) induced by TLR2 or TLR4 are markedly enhanced due to the lack of IRF4-mediated negative regulation in the presence of CD-associated *NOD2* mutations.

The activation of NOD2 mediates tolerogenic responses against the intestinal microbiota in macrophages and DCs, thereby inhibiting the development of chronic intestinal inflammation (8). Previous studies have elucidated some of the molecular mechanisms underlying the downregulation of proinflammatory responses against intestinal bacteria by focusing on the crosstalk between NOD2 and TLRs. As mentioned above, MDP is a degradation product of PGN in the bacterial cell wall (3, 8). PGN activates TLR2 independently of NOD2, suggesting a crosstalk between NOD2 and TLR2 (**Figure 3A**). Previous studies, including ours, initially found that the simultaneous stimulation of the TLR2 and NOD2 pathways reduces the production of proinflammatory T helper type 1 (Th1) cytokines in human and murine macrophages and DCs when compared with that of those stimulated by the TLR2 pathway alone (28, 41–45). This NOD2-mediated downregulation of proinflammatory cytokine responses against TLR2 is accompanied by reduced nuclear translocation of NF-κB subunits, including p65, p50, and c-Rel (41, 43, 44). NOD2-mediated negative regulation of TLR2-mediated Th1 responses, which was initially reported in *in vitro* studies, was also observed in an *in vivo* colitis model. We established a bacterial antigen-specific colitis model induced by adoptive transfer of ovalbumin (OVA)-specific CD4^+^ T cells, followed by the intrarectal administration of *Escherichia coli* expressing OVA (42). This bacterial OVA-specific colitis model is driven by OVA-specific Th1 responses. NOD2-deficient mice are more susceptible to bacterial OVA-specific colitis, when compared with NOD2-intact mice, and are characterized by the excessive accumulation of OVA-specific Th1 cells in the colonic lamina propria (42). Notably, severe bacterial OVA-specific colitis in NOD2-deficiency relies on the activation of TLR2 because mice double deficient in NOD2 and TLR2 display markedly less colonic inflammation and accumulation of OVA-specific Th1 cells in the colonic lamina propria (42). Intrarectal administration of 50% ethanol and PGN causes TLR2-dependent experimental colitis, characterized by a Th1 response (46). Consistent with the data obtained from NOD2-deficient mice, NOD2-transgenic mice under the control of a major histocompatibility complex class II promoter, that is, mice overexpressing NOD2 in macrophages and DCs, were resistant to TLR2-dependent experimental colitis induced through the intrarectal administration of PGN when compared with wild-type mice (43). Resistance to TLR2-dependent colitis in NOD2-transgenic mice is associated with a diminished Th1 response (43). The data obtained from bacterial OVA-specific or TLR2-dependent colitis model utilizing NOD2-deficient and NOD2-transgenic mice provide evidence that the crosstalk between NOD2 and TLR2 contributes to the maintenance of intestinal homeostasis and that NOD2 functions as a negative regulator of TLR2-mediated colitogenic Th1 responses. However, TLRs that induce proinflammatory cytokine responses against intestinal bacteria are not limited to TLR2 (47). Therefore, the NOD2-mediated negative regulation of TLR2 alone cannot explain the immunopathogenesis of CD in the presence of *NOD2* mutations.

It is well-established that prior exposure of macrophages and DCs to lipopolysaccharide (LPS) causes these cells to become refractory to subsequent challenge with a broad range of TLR ligands, not limited to TLR4 ligands (48). This phenomenon, known as endotoxin tolerance, is associated with immunosuppression in sepsis (48). As in the case of endotoxin tolerance, prior exposure of DCs to MDP reduces the production of proinflammatory cytokines upon subsequent challenge with multiple TLR ligands. Pre-stimulation of murine and human DCs with MDP markedly reduces the production of NF-κB-dependent proinflammatory cytokines, including IL-6, IL-12, and TNF-α, upon subsequent challenge with TLR2, TLR3, TLR4, TLR5, and TLR9 ligands (**Figure 3A**) (28). The phenomenon of MDP tolerance has been confirmed in several studies; accordingly, a lack of MDP tolerance has been suggested to impact CD immunopathogenesis in the presence of loss-of-function *NOD2* mutations (8, 49, 50). This notion is strongly supported by studies on dextran sodium sulfate (DSS)-or trinitrobenzene sulfonic acid (TNBS)-induced colitis. Prior systemic injection of MDP protects NOD2-intact mice from TNBS-induced colitis, whose effects are accompanied by diminished NF-κB activation and proinflammatory cytokine responses against multiple TLR ligands in the colonic lamina propria immune cells (28). Similarly, systemic injection of MDP during the initial phases of DSS consumption also protects NOD2-intact, but not NOD2-deficient, mice from DSS-induced colitis, whose effects are also associated with markedly reduced NF-κB activation and proinflammatory cytokine responses against multiple TLR ligands in the colonic lamina propria immune cells (26, 28). Taken together, these *in vitro* and *in vivo* studies support the idea that MDP tolerance mediated by intact NOD2 signaling contributes to the generation of tolerogenic immune environments against the gut bacteria.

Regarding the molecular mechanisms accounting for MDP tolerance, previous studies, including ours, have identified IRF4 as a critical effector molecule involved in the NOD2-mediated suppression of TLR signaling (**Figure 3A**) (27–29, 35, 51, 52). IRF4 functions as a prototypical negative regulator of NF-κB activation induced by TLRs signaling, as revealed by the fact that the macrophages and DCs isolated from IRF4-deficient mice display enhanced proinflammatory cytokine production upon stimulation with TLR4 or TLR9 ligands (53, 54). Intriguingly, MDP activation of NOD2 markedly enhances IRF4 expression in DCs, thereby suppressing the NF-κB-mediated proinflammatory cytokine responses (27–29, 35, 51, 52). Mechanistically, IRF4 induced by NOD2 interacts with RIPK2, TRAF6, and myeloid differentiation factor 88 (MyD88) and subsequently inhibits nuclear translocation of NF-κB subunits (27, 28). Given that TRAF6 is one of the E3 ligases of RIPK2, it is likely that IRF4 downregulates the polyubiquitination of RIPK2. Indeed, IRF4 activation induced by NOD2 inhibits K63-linked, but not K48-linked, polyubiquitination of RIPK2 by binding to the kinase domain (KD) and intermediate domain of RIPK2 (27). In addition, ATG16L1 acts cooperatively with IRF4 to inhibit the K63-linked polyubiquitination of RIPK2 by binding to its KD (35). Collectively, these studies suggest that the activation of IRF4 and ATG16L1 as a result of MDP recognition by NOD2 inhibits the TLRs-mediated NF-κB activation and subsequent proinflammatory cytokine responses through the inhibition of K63-linked polyubiquitination on RIPK2 (**Figure 3A**). On the contrary, DCs bearing CD-associated *NOD2* mutations exhibit enhanced TLRs-mediated NF-κB activation and subsequent colitogenic cytokine responses due to the lack of activation of IRF4 and ATG16L1, both of which inhibit the K63-linked polyubiquitination on RIPK2 (**Figure 3B**). IRF4 also downregulates K63-linked, but not K48-linked polyubiquitination of TRAF6 whereas regulation of MyD88 polyubiquitination by NOD2 has not been clarified (27).

The NOD2-IRF4 axis plays a protective role in the development of experimental colitis. MDP-mediated activation of NOD2 inhibits the DSS- or TNBS-induced colitis via suppression of NF-κB-dependent proinflammatory cytokine responses. MDP-induced protection requires intact IRF4 signaling, as IRF4-deficient mice are not protected from DSS-induced colitis despite the MDP-mediated activation of NOD2 (28). In addition, the development of TNBS-induced colitis is markedly suppressed in mice overexpressing IRF4, whose effects are associated with the downregulation of NF-κB-dependent proinflammatory cytokine responses (27, 29). Negative regulation of TLRs-mediated proinflammatory cytokine responses by the NOD2-IRF4 axis also suppresses colorectal tumorigenesis, obesity-induced insulin resistance, and Blau syndrome, suggesting that this pathway is crucial for the maintenance of immune homeostasis not only in the gut but also in adipose tissues and joints (51, 52, 55). Taken together, accumulating evidence supports the notion that MDP-mediated activation of NOD2 arbitrates tolerogenic responses against the TLR ligands derived from intestinal bacteria through the inhibition of K63-linked polyubiquitination of RIPK2 via the induction of IRF4 expression.

Finally, it is worth noting that NOD2 activation can enhance IL-12 and Th1 responses mediated by LPS activation of TLR4 (56, 57). Such discrepancy can be explained by the doses of LPS tested; MDP activation of NOD2 negatively and positively regulates TLR4-mediated Th1 responses when LPS doses are high and low, respectively (56, 57). Synergic activation of NOD2 and TLR4 seen in low magnitude of TLR4 signaling may contribute to the host defense against invading gut bacteria. On the contrary, negative regulatory function of NOD2 on TLR4 seen in high magnitude of TLR4 signaling may be indispensable for the maintenance of intestinal homeostasis.

### 3.2 NOD2-mediated negative regulation on the production of IRF3 or IRF7-dependent type I IFNs triggered by TLRs

Double-stranded DNA derived from intestinal bacteria activates TLR9, followed by the robust production of type I IFNs by macrophages and DCs (58). However, whether type I IFN responses induced by the activation of TLR9 play protective or pathogenic roles in IBD remains controversial. TLR9 activation prior to the initiation of DSS drinking prevents the development of DSS-induced colitis in a type I IFN-dependent manner (59, 60). Indeed, mice deficient in the type I IFN receptor are more susceptible to DSS-induced colitis than type I IFN receptor-intact mice (61). Mechanistically, type I IFNs have been shown to promote mucosal tissue repair by inducing amphiregulin (61). In contrast to these reports showing the protective roles of the TLR9-type I IFN axis in experimental colitis, Obermeier et al. provided evidence that TLR9 activation during the induction phase of DSS drinking exacerbates DSS-induced colitis through the induction of proinflammatory cytokine responses (62–64). Consistent with this pathogenic role of type I IFNs induced by TLR9, the active colonic mucosa of patients with UC and CD displays enhanced type I IFN signaling pathways (65, 66). The effects of the crosstalk between TLR9 and NOD2 on the maintenance of intestinal homeostasis are poorly understood; however, MDP-mediated activation of NOD2 acts synergistically with TLR9 ligands to promote IL-12 production by human DCs (57).

**Figure 4:**
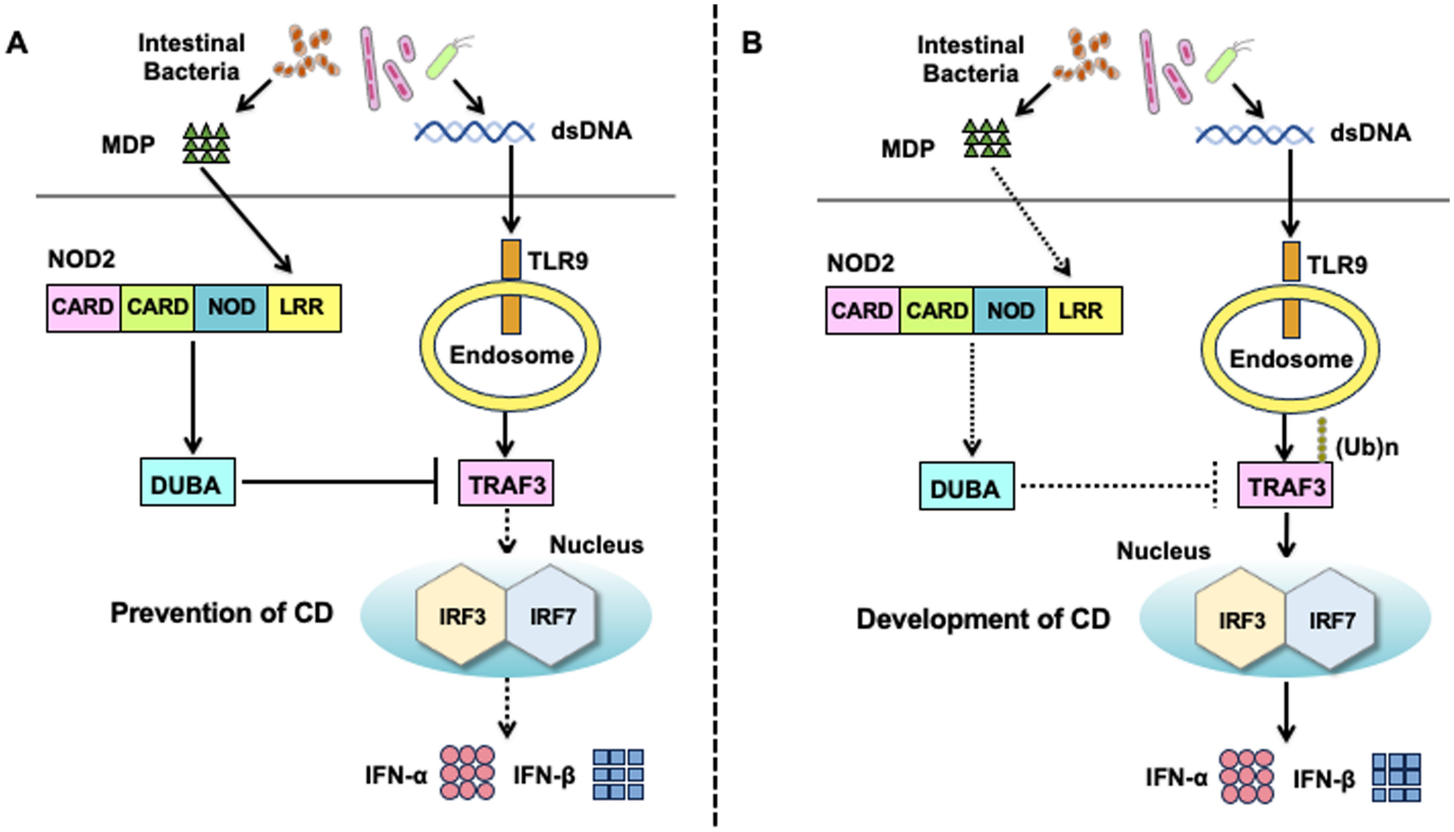
Molecular mechanisms underlying the negative regulation on toll-like receptor 9-mediated type I IFN responses by the activation of nucleotide-binding oligomerization domain 2. **(A)** Molecular mechanisms underlying the negative regulation on toll-like receptor 9 (TLR9)-mediated type I IFN responses in the presence of intact nucleotide-binding oligomerization domain 2 (NOD2). Muramyl dipeptide (MDP) activation of NOD2 enhances the expression of deubiquitinating enzyme A (DUBA). DUBA induced by the activation of NOD2 inhibits the K63-linked polyubiquitination of TNF receptor-associated factor 3 (TRAF3) to downregulate the nuclear translocation of interferon regulatory factor 3 (IRF3) and IRF7, both of which are required for optimal type I IFN responses mediated by TLR9. Downregulation of IRF3/7-dependent type I IFN responses contributes to the maintenance of tolerogenic responses toward intestinal bacteria, thereby inhibiting the development of Crohn’s disease (CD). **(B)** Molecular mechanisms underlying CD development in the presence of *NOD2* mutations. CD-associated *NOD2* mutations are loss-of-function mutations that fail to recognize MDP. DUBA expression is not induced in the presence of CD-associated *NOD2* mutations. CD-associated *NOD2* mutations augment type I IFN responses induced by TLR9 owing to a lack of DUBA-mediated negative regulation. Excessive production of type I IFNs leads to the development of CD.

To clarify the role of the crosstalk between NOD2 and TLR9, we initially examined the production of IFN-α by human peripheral blood monocytes and plasmacytoid dendritic cells (pDCs) stimulated with NOD2 and TLR9 ligands (67, 68). pDCs constitute a specialized DC population with the ability to produce large amounts of IFN-α (69). Production of IFN-α by monocytes and pDCs was much lower in the cells co-stimulated with MDP and CpG (a TLR9 ligand, double-stranded DNA) than in those stimulated with CpG alone, suggesting that NOD2 functions as a negative regulator of TLR9-induced type I IFN responses (**Figure 4A**) (67, 68). MDP-mediated activation of NOD2 suppresses the nuclear translocation of IRF3 and IRF7, resulting in reduced transcription of type I IFN genes. K63-linked polyubiquitination of TRAF3 is an indispensable step for the production of type I IFNs induced by TLR9 (70). Deubiquitinating enzyme A (DUBA) selectively cleaves K63-linked polyubiquitin chains on TRAF3 (71). Regarding the molecular mechanisms accounting for NOD2-mediated negative regulation on type I IFN responses induced by TLR9, we found that co-activation with NOD2 and TLR9 markedly upregulates DUBA expression and consequently reduces IFN-α production through the suppression of K63-linked polyubiquitination on TRAF3 (67, 68). As mentioned above, CpG-mediated activation of TLR9 during the initial phase of DSS-drinking exacerbates DSS-induced colitis (62–64). This exacerbation of DSS-induced colitis by the CpG-mediated activation of TLR9 was dependent on type I IFN signaling pathways, as mice deficient in the type I IFN receptor did not display aggravation of DSS colitis (67, 68).

We then turned our attention to the role of the crosstalk between NOD2 and TLR9 in experimental colitis. Notably, the severity of DSS-induced colitis was much lower in mice treated with co-intraperitoneal injection of MDP and CpG than in those treated with CpG alone. The suppression of TLR9-induced exacerbation of DSS-induced colitis was associated with the downregulation of type I IFN and Th1 responses in the colon. Thus, MDP-mediated activation of NOD2 prevents the exacerbation of DSS-induced colitis induced by TLR9 activation (67, 68). Importantly, attenuation of DSS-induced colitis by NOD2 activation was accompanied by decreased and increased expression of type I IFNs and DUBA, respectively, in the colon (67, 68). Indeed, the blockade of DUBA-mediated deubiquitination of TRAF3 by DUBA-specific siRNA cancelled the negative regulatory effects of NOD2 on DSS-induced colitis in mice treated with CpG, and mice treated with DUBA-siRNA displayed severe DSS-induced colitis even after repeated MDP injections (67, 68). Collectively, these studies suggest that the recognition of MDP derived from intestinal bacteria by NOD2 results in the inhibition of colitogenic type I IFN and Th1 responses induced by the commensal DNA activation of TLR9 (72). Intact NOD2 plays a negative regulatory function in TLR9-induced type I IFN responses by upregulating DUBA expression (67, 68). In the presence of CD-associated *NOD2* mutations, impaired recognition of MDP results in inefficient induction of DUBA, which leads to excessive and colitogenic type I IFN and Th1 responses upon TLR9 activation (**Figure 4B**).

One question that arises from these studies is how co-stimulation with NOD2 and TLR9 ligands induces DUBA expression. In this regard, IL-1β signaling might be involved in the regulation of DUBA expression. Defective IL-1 receptor signaling increases DUBA expression, leading to the suppression of type I IFN production and K63-linked polyubiquitination of TRAF3 (73). Therefore, it may be possible that the downregulation of proinflammatory IL-1β production by NOD2 results in the upregulation of DUBA expression and contributes to the maintenance of intestinal homeostasis. Finally, it is worth mentioning that DUBA plays pathogenic rather than protective roles in the development of TNBS-induced colitis (74). Knockdown of DUBA expression by its antisense oligonucleotides reduces TNF-α production by colonic lamina propria mononuclear cells isolated from mice challenged with intrarectal TNBS administration (74). The pathogenic or protective roles of DUBA in experimental colitis need to be examined in other types of colitis models, including IL-10-deficient mice and T cell transfer models (75, 76).

### 3.3 Therapeutic targets of CD and UC in the NOD2 signaling pathways

**Figure 5:**
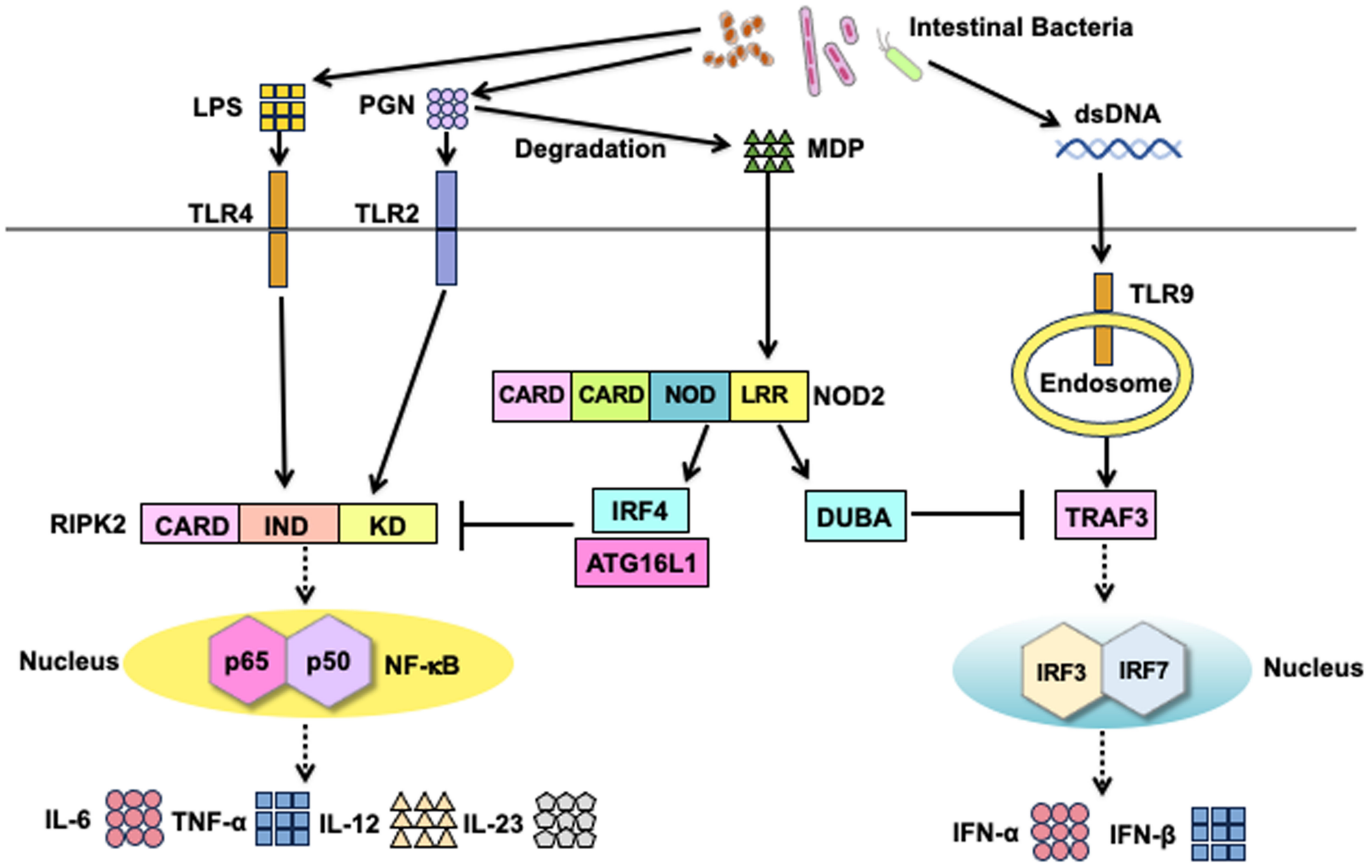
Therapeutic targets of Crohn’s disease and ulcerative colitis in the NOD2 signaling pathways. Possible therapeutic approaches for the clinical application of NOD2 signaling pathways in Crohn’s disease (CD) and ulcerative colitis (UC). Muramyl dipeptide (MDP)-mediated activation of NOD2 enhances the expression of interferon regulatory factor 4 (IRF4) and deubiquitinating enzyme A (DUBA) to suppress the nuclear translocation of nuclear factor-κB (NF-κB) subunits and IRF3/7, respectively. IRF4 acts synergistically with autophagy-related 16 like 1 (ATG16L1) to inhibit polyubiquitination of receptor-interacting serine/threonine protein kinase 2 (RIPK2), thereby suppressing the production of NF-κB-dependent colitogenic cytokines, such as IL-6, IL-12, IL-23, and TNF-α. DUBA inhibits polyubiquitination on TNF receptor-associated factor 3 (TRAF3) to downregulate type I IFN responses. Inhibition of RIPK2 activation is a promising therapeutic approach for CD and UC. Enhancement of IRF4, ATG16L1, and DUBA expression may also be useful in the treatment of CD and UC. Intestinal bacteria, which can produce large amounts of MDP, may be promising probiotics.

The anti-inflammatory function of the MDP-NOD2 axis was tested in experimental colitis and human IBD samples for clinical application (**Figure 5**). Given the potent anti-inflammatory activity of MDP, it is likely that the MDP-rich gut mucosa is resistant to IBD. Gao et al. revealed that *Firmicutes*-derived DL-endopeptidase can generate large amounts of MDP in the gut and that the activity of this enzyme decreases in patients with CD (77). In addition, fecal microbiota of CD patients with low DL-endopeptidase predisposes mice to DSS-induced colitis through the upregulation of IL-6, TNF-α, and RIPK2 (77). In subsequent studies, the same research group identified an uncharacterized secreted protein (called LPH) from *Lactobacillus* (78). LPH is a bifunctional PGN hydrolase composed of DL-endopeptidase and N-acetyl-β-D-muramidase, with the ability to degrade PGN into MDP. LPH administration protects mice from TNBS-induced colitis in an NOD2-dependent manner (78). Similarly, another report showed that oral administration of selected lactobacilli with the ability to produce large amounts of MDP inhibited the development of TNBS-induced colitis through the downregulation of IL-1β and upregulation of IL-10 (79). Given these results, probiotic approaches utilizing MDP-NOD2 signaling are currently under development.

The RIPK2 signaling complex is composed of E3 ligases (cIAP1, cIAP2, TRAF6, XIAP, and pellino3), RIPK2, and TAK1 (12). We examined the mRNA expression of components of the RIPK2 signaling complex (80) and found that the mRNA expression of *RIPK2, cIAP1, cIAP2, TRAF6*, and *TAK1* was significantly higher in the colonic mucosa of patients with UC and CD than in that of the healthy controls (80). In addition, the mRNA expression of *RIPK2, cIAP1, cIAP2, TRAF6*, and *TAK1* showed a trend similar to that of IL-6, TNF-α, and IL-12p40 (80). At the protein level, the intensity of molecular interactions between RIPK2 and cIAP2 or TAK1 corresponds to the expression of IL-6 or TNF-α in the active colonic mucosa of patients with UC or CD (80). Consistent with these data obtained in human IBD samples, knockdown of RIPK2 by its specific siRNA protects mice from DSS-induced colitis, whose effects are accompanied by reduced production of IL-6 and TNF-α against TLR ligands in the colonic lamina propria immune cells (80). Finally, the administration of RIPK2 siRNA protected NOD1- or NOD2-deficient mice from DSS-induced colitis, suggesting that RIPK2, activated by TLR2 and/or TLR4, plays a colitogenic role. These studies, employing experimental colitis and human IBD samples, have facilitated the development of RIPK2 inhibitors for IBD treatment. Indeed, various RIPK2-specific inhibitors have been successfully developed (81–83). These inhibitors downregulate the production of proinflammatory cytokines in human IBD biopsy samples and display potent therapeutic effects against DSS-induced colitis (81–83). The verification of RIPK2 as a promising target for IBD requires further studies utilizing genetically engineered RIPK2-deficient mice, as knockdown of gene expression by siRNAs could cause non-specific off target effects.

As mentioned above, NOD2 activation inhibits the TLRs-mediated colitogenic responses by inducing IRF4 (27–29, 35, 51, 52). Another approach for the treatment of IBD is the activation of IRF4. Theoretically, DC-specific activation of IRF4 is assumed to inhibit the development of IBD by downregulating proinflammatory cytokine responses against TLR ligands derived from intestinal bacteria (27–29, 35, 51, 52). However, caution should be exercised regarding the clinical application of IRF4 activation in IBD, as IRF4 expressed in CD4^+^ T cells mediates the development of Th17-dependent experimental colitis (84, 85). DC-specific activation of IRF4 is required for its clinical application in IBD.

The active colonic mucosa of patients with CD and UC is characterized by the upregulation of IFN-stimulated gene (ISG), regardless of the *NOD2* mutation status (65–68). The MDP-NOD2 axis downregulates TLR9-induced ISG by upregulating DUBA expression (67, 68). *DUBA* mRNA expression was reported to be significantly higher in patients with remitted CD than in those with active CD (67, 68). Therefore, small molecules that induce DUBA activation may be useful in the treatment of CD. However, to the best of our knowledge, small molecules that can regulate DUBA function have not yet been identified.

## 4 Conclusions

In this review, we focused on the negative regulatory function of NOD2 in TLRs-mediated colitogenic and proinflammatory cytokine responses. It should be noted, however, that NOD2 has diverse immunological functions in the maintenance of intestinal homeostasis. For example, within the intestinal crypt, Lgr5^+^ stem cells constitutively express NOD2, and MDP recognition by stem cell NOD2 initiates the program of gut epithelial regeneration (86). In addition, macrophages bearing CD-associated *NOD2* mutations create the aberrant macrophage-fibroblast interaction characterized by the gp130-mediated excessive production of IL-6, IL-11, and oncostatin M (87). Identification and characterization of novel NOD2 functions provide new insights into the molecular mechanisms of CD owing to the presence of *NOD2* mutations and may lead to the development of novel treatments for IBD patients with intact or mutated *NOD2* genes.
